# A machine learning model to predict the risk of depression in US adults with obstructive sleep apnea hypopnea syndrome: a cross-sectional study

**DOI:** 10.3389/fpubh.2023.1348803

**Published:** 2024-01-08

**Authors:** Enguang Li, Fangzhu Ai, Chunguang Liang

**Affiliations:** Department of Nursing, Jinzhou Medical University, Jinzhou, China

**Keywords:** machine learning, depression, OSAHS, prediction models, NHANES

## Abstract

**Objective:**

Depression is very common and harmful in patients with obstructive sleep apnea hypopnea syndrome (OSAHS). It is necessary to screen OSAHS patients for depression early. However, there are no validated tools to assess the likelihood of depression in patients with OSAHS. This study used data from the National Health and Nutrition Examination Survey (NHANES) database and machine learning (ML) methods to construct a risk prediction model for depression, aiming to predict the probability of depression in the OSAHS population. Relevant features were analyzed and a nomogram was drawn to visually predict and easily estimate the risk of depression according to the best performing model.

**Study design:**

This is a cross-sectional study.

**Methods:**

Data from three cycles (2005–2006, 2007–2008, and 2015–2016) were selected from the NHANES database, and 16 influencing factors were screened and included. Three prediction models were established by the logistic regression algorithm, least absolute shrinkage and selection operator (LASSO) algorithm, and random forest algorithm, respectively. The receiver operating characteristic (ROC) area under the curve (AUC), specificity, sensitivity, and decision curve analysis (DCA) were used to assess evaluate and compare the different ML models.

**Results:**

The logistic regression model had lower sensitivity than the lasso model, while the specificity and AUC area were higher than the random forest and lasso models. Moreover, when the threshold probability range was 0.19–0.25 and 0.45–0.82, the net benefit of the logistic regression model was the largest. The logistic regression model clarified the factors contributing to depression, including gender, general health condition, body mass index (BMI), smoking, OSAHS severity, age, education level, ratio of family income to poverty (PIR), and asthma.

**Conclusion:**

This study developed three machine learning (ML) models (logistic regression model, lasso model, and random forest model) using the NHANES database to predict depression and identify influencing factors among OSAHS patients. Among them, the logistic regression model was superior to the lasso and random forest models in overall prediction performance. By drawing the nomogram and applying it to the sleep testing center or sleep clinic, sleep technicians and medical staff can quickly and easily identify whether OSAHS patients have depression to carry out the necessary referral and psychological treatment.

## Introduction

Depression is a widespread mental health disorder that seriously limits the patient’s psychological and social function, which reduces their quality of life. At the same time, depression also brings severe financial and emotional stress to the families of patients. Its main features include persistent fatigue, depression, low mood, reduced interest, and poor concentration (1). Depression is related to mental health and is now the main reason for the global burden of disease. In addition to the severe influence on personal emotion and psychological state, it may seriously impact work and personal relationships (2). In 2015, the WHO announced that, globally, depression affects more than 300 million people, or 4.4% of the world’s population, and is the leading cause of disability globally (3), with about 1 million people dying of depression each year (4). At the same time, depression also imposes significant socioeconomic costs. The annual cost of treating depression in the US is reported to be as high as $210 billion (5). However, in high-income countries, nearly half of people with depression are not diagnosed or treated. In low and middle-income countries, the proportion is as high as 80%–90%. Early detection and prevention of depression is, therefore, essential to reduce the global burden. Society needs to take action early in life and in adversity and the impact of inequality (6).

Obstructive Sleep Apnea Hypopnea Syndrome (OSAHS) is a chronic disease characterized by recurrent upper airway collapse and obstruction during sleep (7), resulting in periodic reduction or cessation of ventilation, which causes hypoxia, hypercapnia and sleep arousal (8). Cross-sectional studies have found that OSAHS may increase the risk of depression (9). In addition, a dose–response relationship has been found between the severity of OSAHS and the risk of depression (10). That is, the more severe the OSAHS, the higher the risk of depression. This means that the presence of OSAHS may cause more significant difficulties in the treatment of depression. A cross-sectional study of community and clinical populations found a relatively high prevalence of depression in patients with OSAHS of 17% (11). In contrast, the prevalence of depression among patients with a definite diagnosis of OSAHS in the sleep clinic showed a wide variability, ranging from 5% to 63% (2). To verify the causal relationship between OSAHS and depression, according to a prospective longitudinal study of depression a year later with OSAHS between cause and effect (12). Therefore, we suggest that screening for psychiatric in patients with OSAHS timely find depression to effectively prevent and treat depression and reduce the impact on the quality of life and social economy.

Currently, one of the early screening methods for depression is to determine the presence or absence of depression by using the Depression Self-Rating Scale (DSRS). However, there is no self-assessment scale for depression for patients with OSAHS. Although several self-rating depression scales have been shown to have reliable reliability and validity in OSAHS patients (13–16), these scales still do not accurately predict the risk of depression in OSAHS patients. Clinical predictive modeling was introduced to solve this problem. It is a mathematical formula to estimate the probability that a particular individual is currently suffering from a disease or experiencing a specific outcome. In this study, a prediction model was used to estimate the likelihood of depression in OSAHS patients to more accurately assess the risk of depression in patients and take appropriate interventions.

However, traditional statistical methods are only suitable for solving simple linear problems rather than for dealing with complex nonlinear relationships. Secondly, traditional models lack adaptive learning capabilities and require manual selection and extraction of variables. This process requires specialized knowledge and experience and relies on prior knowledge or specific rules to build and adapt. Moreover, the traditional model can only deal with small-scale data sets, and the processing effect on high-dimensional data is poor (17).

Therefore, introducing machine learning (ML), a powerful and intelligent tool, can solve all these problems. ML models can adaptively learn and adjust models from data without manually specifying model parameters or rules. In addition, ML models also have good generalization ability. It can effectively generalize the patterns learned from the training data to the new data. In addition, ML models can handle large-scale data, automatically extract features, and build models from the data. Finally, the ML model also has high interpretability, can through the way of visualization and explanatory, help researchers to understand the behavior of the model and the decision-making process (18, 19).

At present, the ML has been widely applied in the depression risk prediction model was constructed. For example, Dai Su et al. used ML algorithms to construct a risk prediction model for depression in older Chinese adults (20). Fang Xia et al. developed a prediction model for depression caused by heavy metals in older people using the ML method based on the National Health and Nutrition Examination Survey (NHANES) database (21). The research result shows that ML, which improves the prediction accuracy of depression, reduces error and mass data processing, and so on, shows great potential.

After a comprehensive literature search, we found that most of the previous studies focused on exploring the correlation between OSAHS and depression. At the same time, there are a large number of predictive studies of patients with OSAHS (9, 22–26). However, no studies have been found to predict whether patients with OSAHS will develop depression. This includes studies using traditional statistical methods (such as logistic regression) and ML methods (such as random forest, SVM, etc.) to construct depression risk prediction models for OSAHS patients. Therefore, this study selected a large data sample from the NHANES database and screened for influencing factors associated with depression. To construct a risk prediction model that can predict whether OSAHS patients will have depression using ML methods.

## Materials and methods

### Data and sample

#### Description of National Health and Nutrition Examination Survey data

The data used in this study come from the NHANES database published by the Centers for Disease Control and Prevention (CDC). NHANES, a population-based cross-sectional survey, aims to collect information about relevant American adults’ and children’s diet, nutrition, health, and health behavior (5). A representative sample of households across the United States was selected using multistage stratified random sampling. Since 1999, the NHANES program has conducted a nationally representative sample every two years. Each year, NHANES investigators conduct home visits and in-person interviews with a nationally representative sample of about 5,000 people of all ages. They collect data on basic information, family structure, health status, and eating habits of the respondents. After the face-to-face survey, participants were invited to a temporary examination center for various physical measurements, physical function tests, and laboratory tests. Finally, the collected data will be collated, coded, and anonymized before being stored in the NHANES database. It was also approved by the Research Ethics Review Board of the National Center for Health Statistics (NCHS). Each participant was asked to sign a consent form, including all the questionnaires, and check. For participants younger than 18 years of age, they were required to complete data collection with informed consent from their parents or guardians (27, 28). In this study, the OSAHS population was selected based on participants’ self-report of the question “How often do you snort/stop breathing?” on the sleep questionnaire. Therefore, we excluded data periods that did not include this question in the sleep questionnaire and finally selected data from the three 2-year periods that had this question (2005–2006, 2007–2008, and 2015–2016). These data will be used to construct a prediction model for depression in the OSAHS population. All data were downloaded from the official NHANES website.^1^

### Outcome variable

The 9-item Patient Health Questionnaire-9 (PHQ-9) was used in this study to assess depression in patients with OSAHS. The questionnaire used a four-point Likert scale, with options for each item including 0 (not at all), 1 (a few days), 2 (more than half a day), and 3 (almost every day). Each item is scored from 0 to 3, and the total score ranges from 0 to 27 (29). Patients with a PHQ-9 total score ≥ 5 were considered to have depression according to study criteria (30). It is worth noting that the ultimate purpose and significance of this study is to estimate the probability of depression in OSAHS patients by selecting the best prediction model and constructing a nomogram based on the relevant influencing factors. The application of this nomogram in sleep testing centers or sleep clinics can help sleep technicians and medical staff quickly and easily identify whether OSAHS patients have depression and make necessary referrals. Therefore, patients were divided into two groups with and without depression only according to whether they would develop depression. However, Kroenke noted that significant clinical significance was often seen in moderate to severe cases and suggested concomitant antidepressants to improve sleep (31). Therefore, if considering the practical application value of psychiatric clinical practice, it is recommended that future research be able to divide the severity of depression in detail and construct multivariate dependent variable prediction models. Such studies are expected to improve the accuracy and predictive power of the model and thus better provide clinical assistance to psychiatrists.

### Predictor variables

In this study, we categorized the OSAHS population based on participants’ responses to the question “How often do you stop breathing?” on a sleep questionnaire. In answer to this question, we recorded responses of 0 (never) as indicating the non-OSAHS population, while responses of 1 (rarely, 1–2 nights per week), 2 (occasionally, 3–4 nights per week), and 3 (often, five or more nights per week) were defined as indicating the OSAHS population.

Data on demographic characteristics of NHANES from 2005–2006, 2007–2008, and 2015–2016 were selected for this study. Data on age, gender, race, education level, marital status, ratio of family income to poverty (PIR), body mass index (BMI), and sleep hours were included. Of these, we selected adults aged 18 years and older for the study. For race, we categorized them into five categories: Mexican American, Other Hispanic, Non-Hispanic White, Non-Hispanic Black, and Other Race. Education level was categorized into five groups: Less than 9th Grade, 9th–11th Grade, High School Grad/GED or Equivalent, Some College or AA degree, and College Graduate or above. Marital status included married, widowed, divorced, separated, unmarried, and living with a partner. Income status was divided into two categories by using PIR: low-income (PIR ≤ 1.3) and non-low-income (PIR > 1.3). BMI was categorized into four types: underweight (BMI < 18.5), normal weight (18.5–24.99), overweight (25.0–29.99), and obese (BMI ≥ 30.0). Sleep hours were also categorized into three categories: short sleep hours (<7 h), normal sleep hours (7–9 h), and long sleep hours (>9 h).

Lifestyle variables include smoking and alcohol drinking. Smoking status was determined based on respondents’ self-reports to two questions: “Have you ever smoked more than 100 cigarettes in your lifetime?” and “Do you currently smoke?.” Smoking status was categorized into three categories: never smoker (lifetime never smoked more than 100 cigarettes, current never smoked), former smoker (lifetime smoked more than 100 cigarettes, current never smoked), and now smoker (lifetime smoked more than 100 cigarettes, current daily smoker or current smoker for a few days).

Alcohol drinking was determined by self-report of respondents on the following questions: “In the past 12 months, how often did you drink any type of alcoholic beverage (measured in days)?” Based on their responses, we defined drinking as three types: never drinking (0), low drinking (1–36 days), and heavy drinking (≥37 days).

Health information variables included general health condition, hypertension, diabetes, asthma, coronary heart disease, and OSAHS severity. General health condition was determined by self-report of respondents on the following questions: “I have some general questions about your health.” and “Would you say your health in general is?” Of these, “excellent,” “very good,” and “good” responses were redefined as “good general health condition.” “Fair” is defined as “General health.” “Poor” is defined as “bad general health condition.”

The presence of the four conditions, hypertension, diabetes, asthma, and coronary heart disease, was determined using a “yes” or “no” response. For diabetes problems, it is essential to note that if respondents answer “Border,” it has also been defined as no diabetes.

OSAHS severity was determined by the subject’s response to the question, “How often do you snort/stop breathing?.” Specifically, “1–2 nights per week” was defined as “mild,” and “3–4 nights per week” was described as “moderate.” “5 or more nights per week” was defined as “severe.” Table 1 provides details of the assignment of each influence factor.

**Table 1:** Predictor variable assignment.

Predictive factors	Variable type	Assignment
Gender	Categorical variables	“Male” = 1, “Female” = 2
Age	Continuous variables	Original value entry
Race	Categorical variables	“Mexican American” = 1, “Other Hispanic” = 2， “Non-Hispanic White” = 3 ， “Non-Hispanic Black” = 4 ，“Other Race” = 5
Education level	Categorical variables	“Less than 9th grade” = 1, “9-11th grade” = 2， “High school graduate” = 3，“Some college or AA degree” = 4 ，“College graduate or above” = 5
Marital status	Categorical variables	“Married” = 1, “Widowed” = 2，“Divorced” = 3, “Separated” = 4，“Never married” =5, “Living with a partner” = 6
PIR	Categorical variables	“Low-income” = 1, “Non-low-income” = 2
General health condition	Categorical variables	“Good” = 1, “General” = 2，“Bad” = 3
Sleep hours	Categorical variables	“Short” = 1, “Normal” = 2，“Long” = 3
BMI	Categorical variables	“Underweight” = 0, “Normal weight” = 1， “Overweight” = 2, “Obese” = 3
Alcohol drinking	Categorical variables	“Never drinking” = 0, “Small amount” = 1， “Large amount” = 2
Smoking	Categorical variables	“Never smoker” = 0, “Former smoker” = 1， “Now smoker” = 2
Hypertension	Categorical variables	“Yes” = 1, “No” = 2
Diabetes	Categorical variables	“Yes” = 1, “No” = 2
Asthma	Categorical variables	“Yes” = 1, “No” = 2
Coronary heart disease	Categorical variables	“Yes” = 1, “No” = 2
OSAHS severity	Categorical variables	“Mild” = 1, “Moderate” = 2，“Severe” = 3

PIR, ratio of family income to poverty; BMI, body mass index.

### Statistical analysis

#### Data description

Stata 17.0 software was applied to extract and clean the NHANES data, and SPSS 25.0 and R Studio software were used for statistical analysis and description. Measurements that conformed to normal distribution were expressed as M ± SD (Mean ± standard error), and comparisons between groups were made using the independent samples *t*-test. If it did not meet, it was expressed as M (P25, P75), and comparisons between groups were made using the Mann–Whitney U test. Count data were expressed as *n* (%), and comparisons between groups were made using the *χ*^2^ test, with *p* < 0.05 being considered statistically significant.

#### ML models

Before ML model training and evaluation, we used the set.seed(123) function in R studio software to split the dataset into training and validation sets at a 7:3 ratio. The training set is used for training multiple models, while the validation set is used to verify the performance and generalization ability of the model.

#### Logistic regression model

In R Studio software, the functions and commands of the mlr package were used for univariate logistic regression, followed by multiple collinearity diagnoses in SPSS software, and the variables with statistically significant differences were included in multivariate logistic regression for analysis. The final selected variables were used in R Studio software to draw the nomogram and establish the logistic regression prediction model by the plotLearnerPrediction() function.

#### LASSO model

**Figure 1:**
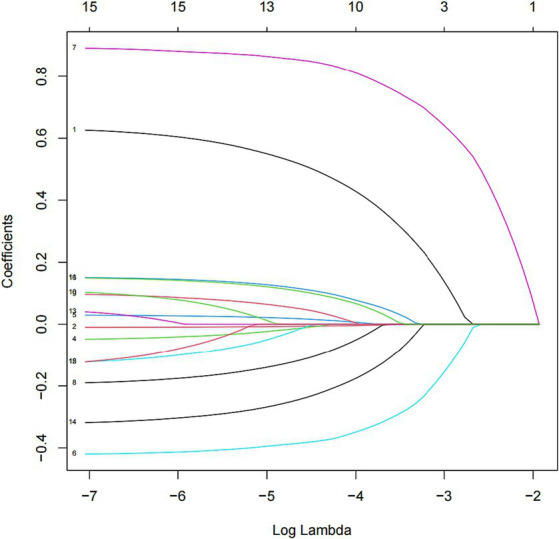
Lasso screening variable dynamic plot.

In this study, we used the mlr package and glmnet package in R Studio software for training and fitting the lasso model. We performed 10-fold cross-validation with the cv.glmnet() function to select the best lambda value. Then, we retrained the lasso model based on the best lambda value and used the coef() function to obtain the coefficients of the model to complete the training of the lasso model. Given that there may be some covariance and correlation between the independent variables, in order to avoid overfitting the model, we performed dimensionality reduction on the independent variables to screen out the influencing factors related to OSAHS depression. Based on the above dimension reduction analysis, the lasso method was used to analyze all the independent variables included in the model, as shown in Figure 1. In this process, the model can be started from the initial to join the independent variable coefficient of compression gradually until the part of the independent variable coefficient is compressed to 0 to avoid the model’s overfitting problem.

#### Random forest model

**Figure 2:**
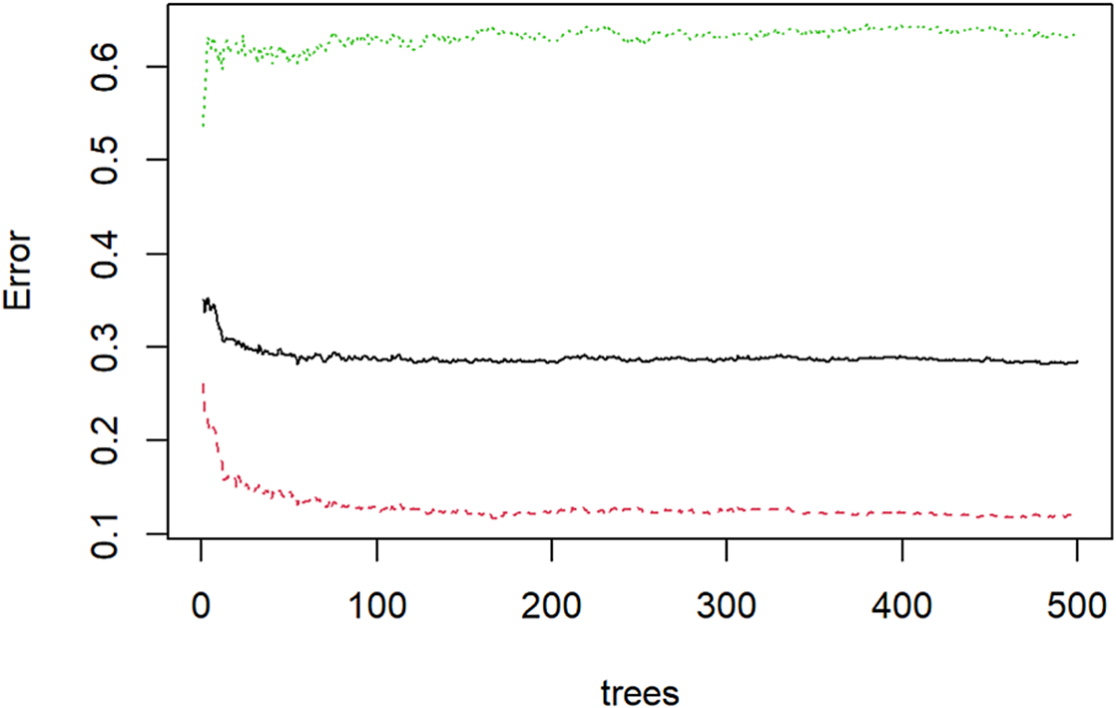
Dynamic relationship between prediction error of random forest and the number of random trees.

Using the randomForest package in the R Studio for training the random forest model. Based on MeanDecreaseGini, We ranked the 16 independent variables and used the random forest feature importance assessment algorithm to derive the importance of each influencing factor (32), selection of important variables with high impact on depression in OSAHS patients. The optimal number of features of the random forest model was chosen according to the out-of-bag error rate. To better understand the relationship between variables and improve the model’s prediction performance. Among the model parameters, there are two key parameters to consider: the number of predicted evaluation indicators (mtry) and the number of random trees (ntree). Among them, mtry is the number of randomly selected evaluation indicators used to construct a random tree, usually the square root of the number of all evaluation indicators in the sample. The tree represents the number of random trees built in the model. When mtry = 5, the minimum error rate outside the package. When ntree = 500, the error is basically stable, and the dynamic relationship between the prediction error of random forest and the number of random trees is shown in Figure 2. Therefore, the parameters of the optimal model are mtry = 5 and ntree = 500. The final selected variables were included in the multivariate logistic regression analysis, and the random forest model was finally constructed.

#### Model comparison

Adopt the receiver operating characteristic curve (ROC curve) and the area under the curve (AUC), specificity, sensitivity, Youden index, and DCA comparison of the model to evaluate and compare the performance of the forecasting model. Specifically, this study used the pROC package of R studio software to draw the ROC curve of the prediction model. Then, calculate the AUC, specificity, sensitivity, and Youden index. Subsequently, the ROC curves of the three models were compared using the DeLong test to judge whether the ROC curves of the three models were significantly different. Finally, the “rmda” package and the “decision_curve” function algorithm were used to draw and compare the differences between the DCA curves of different models.

## Results

### Patient screening and statistical analysis process

**Figure 3:**
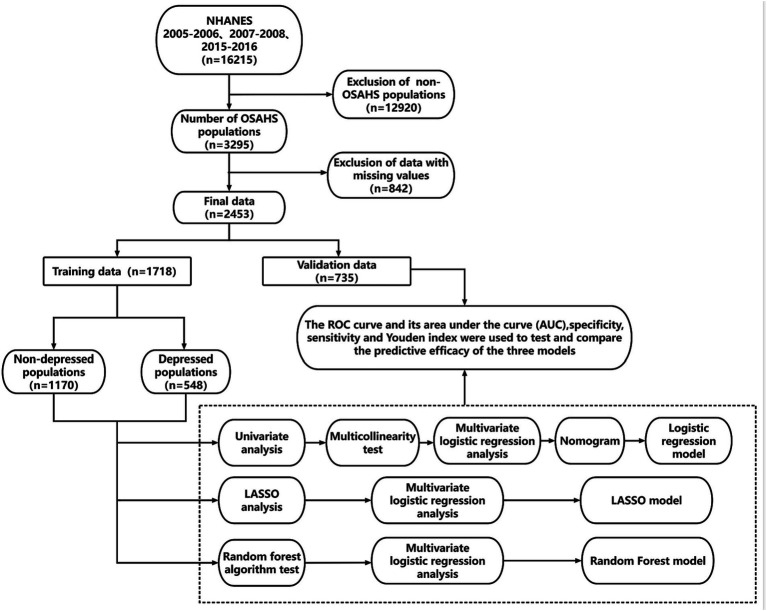
Researchers and statistical flowchart.

After strict data cleaning, we selected the three NHANES data cycles: 2005–2006, 2007–2008, and 2015–2016. In the process, we finally chose to include 2,453 patients in the standard. All eligible patients were randomly divided into a training set and a validation set at a ratio of 7:3, with 1718 patients in the training set and 735 in the validation set. Such a dataset partitioning is consistent with the approach Yalong Zhang et al. adopted in their study of ML prediction models (33). Meanwhile, Jianping Lv et al., in their research, used a ML model designed to predict the risk of bullying victimization among adolescents in the same way that our dataset was partitioned (34). The detailed screening process is shown in Figure 3.

### Comparison of baseline information

Through the statistical analysis, this study found no significant difference in baseline characteristics between the training and validation sets (*p* > 0.05). This shows no deviation between the two groups caused by the uneven distribution of the dependent variable, as shown in Table 2. In addition, we divided the training set into non-depressed and depressed groups and compared baseline information between the two groups. Specific comparative results can be found in Table 3.

**Table 2:** Comparison of baseline data between the two groups.

Predictive factors	Training data (*n* = 1,718)	Validation data (*n* = 735)	*χ*^2^/*t*	*p* value
Age	50.01 ± 15.984	50.35 ± 16.274	−0.471	0.341
Gender			3.835	0.052
Male	1,091(63.5)	436(59.3)		
Female	627 (36.5)	299(40.7)		
Race			5.913	0.206
Mexican American	286(16.6)	129(17.6)		
Other Hispanic	178(10.4)	61(8.3)		
Non-Hispanic White	811(47.2)	328(44.6)		
Non-Hispanic Black	326(19.0)	159(21.6)		
Other race	117(6.8)	58(7.9)		
Education level			3.250	0.517
Less than 9th grade	167(9.7)	63(8.6)		
9–11th grade	263(15.3)	124(16.9)		
High school graduate	426(24.8)	171(23.3)		
Some college or AA degree	528(30.7)	219(29.8)		
College graduate or above	334(19.4)	158(21.5)		
Marital status			5.048	0.410
Married	997(58.0)	435(59.2)		
Widowed	79(4.6)	40(5.4)		
Divorced	183(10.7)	74(10.1)		
Separated	54(3.1)	27(3.7)		
Unmarried	218(12.7)	98(13.3)		
Living with a partner	187(10.9)	61(8.3)		
PIR			0.645	0.422
Low-income	483(28.1)	195(26.5)		
Non-low-income	1,235(71.9)	540(73.5)		
General health condition			3.340	0.188
Good	1,233(71.8)	514(69.9)		
General	360(21.0)	176(23.9)		
Bad	125(7.3)	45(6.1)		
Sleep hours			2.070	0.355
Short sleep hours	652(38.0)	262(35.6)		
Normal sleep hours	962(56.0)	434(59.0)		
Long sleep hours	104(6.1)	39(5.3)		
BMI			1.597	0.660
Underweight	14(0.8)	10(1.4)		
Normal weight	319(18.6)	135(18.4)		
Overweight	548(31.9)	235(32.0)		
Obese	837(48.7)	355(48.3)		
Alcohol drinking			0.334	0.846
Never drinking	398(23.2)	165(22.4)		
Low drinking	1,303(75.8)	564(76.7)		
Heavy drinking	17(1.0)	6(0.8)		
Smoking			1.392	0.499
Never smoker	741(43.1)	335(45.6)		
Former smoker	519(30.2)	208(28.3)		
Now smoker	458(26.7)	192(26.1)		
Hypertension			0.078	0.781
Yes	728(42.4)	307(41.8)		
No	990(57.6)	428(58.2)		
Diabetes			0.015	0.901
Yes	284(16.5)	123(16.7)		
No	1,434(83.5)	612(83.3)		
Asthma			0.130	0.719
Yes	312(18.2)	129(17.6)		
No	1,406(81.8)	606(82.4)		
Coronary heart disease			1.277	0.258
Yes	104(6.1)	36(4.9)		
No	1,614(93.9)	699(95.1)		
OSAHS severity			1.547	0.461
Mild	777(45.2)	339(46.1)		
Moderate	492(28.6)	221(30.1)		
Severe	449(26.1)	175(23.8)		

PIR, ratio of family income to poverty; BMI, body mass index.

**Table 3:** Comparison of baseline data between depression group and non-depression group in training data.

Predictive factors	Non-depression group (*n* = 1,170)	Depression group (*n* = 548)
Age	50.65 ± 16.332	48.65 ± 15.139
Gender		
Male	801(68.5)	290(52.9)
Female	369(31.5)	258(47.1)
Race		
Mexican American	200(17.1)	86(15.7)
Other Hispanic	116(9.9)	62(11.3)
Non-Hispanic White	540(46.2)	271(49.5)
Non-Hispanic Black	222(19.0)	104(19.0)
Other race	92(7.9)	25(4.6)
Education level		
Less than 9th grade	96(8.2)	71(13.0)
9–11th grade	168(14.4)	95(17.3)
High school graduate	283(24.2)	143(26.1)
Some college or AA degree	354(30.3)	174(31.8)
College graduate or above	269(23.0)	65(11.9)
Marital status		
Married	722(61.7)	275(50.2)
Widowed	52(4.4)	27(4.9)
Divorced	114(9.7)	69(12.6)
Separated	29(2.5)	25(4.6)
Never married	126(10.8)	92(16.8)
Living with a partner	127(10.9)	60(10.9)
PIR		
Low-income	259(22.1)	224(40.9)
Non-low-income	911(77.9)	324(59.1)
General health condition		
Good	944(80.7)	289(52.7)
General	189(16.2)	171(31.2)
Bad	37(3.2)	88(16.1)
Sleep hours		
Short sleep hours	407(34.8)	245(44.7)
Normal sleep hours	709(60.6)	253(46.2)
Long sleep hours	54(4.6)	50(9.1)
BMI		
Underweight	6(0.5)	8(1.5)
Normal weight	239(20.4)	80(14.6)
Overweight	385(32.9)	163(29.7)
Obese	540(46.2)	297(54.2)
Alcohol drinking		
Never drinking	260(22.2)	138(25.2)
Low drinking	899(76.8)	404(73.7)
Heavy drinking	11(0.9)	6(1.1)
Smoking		
Never smoker	541(46.2)	200(36.5)
Former smoker	356(30.4)	163(29.7)
Now smoker	273(23.3)	185(33.8)
Hypertension		
Yes	466(39.8)	262(47.8)
No	704(60.2)	286(52.2)
Diabetes		
Yes	174(14.9)	110(20.1)
No	996(85.1)	438(79.9)
Asthma		
Yes	176(15.0)	136(24.8)
No	994(85.0)	412(75.2)
Coronary heart disease		
Yes	63(5.4)	41(7.5)
No	1,107(94.6)	507(92.5)
OSAHS severity		
Mild	560(47.9)	217(39.6)
Moderate	330(28.2)	162(29.6)
Severe	280(23.9)	169(30.8)

PIR, ratio of family income to poverty; BMI, body mass index.

### Models predict performance in depressed patients with OSAHS

#### Logistic regression model

In the training set, we divided 1718 OSAHS patients into depressed and non-depressed groups. Through the single variable analysis, we found a statistically significant (*p* < 0.05) result of 12 factors involved, including gender, age, education level, marital status, PIR, general health condition, BMI, smoking, hypertension, diabetes, asthma, and OSAHS severity. Subsequently, statistically significant variables in the univariate analysis were included in the multicollinearity diagnosis. According to the analysis results, all variance inflation factors (VIF) involved in the binary logistic regression analysis were less than 5, and the tolerance index was greater than 0.1. This indicates that there is no case of multicollinearity between covariates. All variables are included in the logistic model as a predictor. The results of multicollinearity diagnosis are shown in Table 4. Statistically, there is a difference in the univariate analysis, and there is no multicollinearity of a variable in binary logistic regression analysis. Adopting the positive method step by step and likelihood ratio test, the method of removing confounding factors, finally got into the model’s variables. The results showed that gender, general health condition, BMI, smoking, OSAHS severity, age, education level, PIR, and asthma were significant influencing factors for depression in OSAHS patients. Among these influencing factors, Gender, General health condition, BMI, Smoking, and OSAHS severity were identified as independent risk factors for depression in OSAHS patients. The factors associated with depression in univariate and multivariate analyses are shown in Table 5.

**Table 4:** Multi-collinearity analysis results of predictive variables of depression of OSAHS patients.

Coefficients^a^							
	Non-standardized coefficient		Standardized coefficient			Colinearity statistics	
Model	B	Standard error	Beta	*t*	Significance	Allowance	VIF
(Constant)	0.174	0.134		1.295	0.195		
Gender	0.121	0.022	0.125	5.462	0.000	0.960	1.042
Age	−0.002	0.001	−0.070	−2.679	0.007	0.745	1.342
Education level	−0.008	0.009	−0.022	−0.868	0.385	0.817	1.224
Marital status	0.005	0.006	0.022	0.883	0.377	0.828	1.208
PIR	−0.086	0.026	−0.083	−3.339	0.001	0.820	1.219
General health condition	0.194	0.019	0.254	10.079	0.000	0.789	1.267
BMI	0.019	0.014	0.032	1.373	0.170	0.896	1.117
Smoking	0.031	0.013	0.055	2.319	0.021	0.897	1.115
Hypertension	−0.028	0.023	−0.029	−1.176	0.240	0.809	1.236
Diabetes	0.012	0.031	0.010	0.397	0.691	0.832	1.202
Asthma	−0.069	0.028	−0.057	−2.482	0.013	0.962	1.039
OSAHS severity	0.031	0.013	0.055	2.394	0.017	0.945	1.059

PIR, ratio of family income to poverty; BMI, body mass index.

a Dependent variable: depression.

**Table 5:** Factors associated with depression in univariable and multivariable analyses in the training set.

Predictive factors	Univariable model		Multivariable model	
	OR(95%CI)	*p* value	OR(95%CI)	*p* value
Gender	1.93 (1.57，2.38)	<0.001	1.86 (1.48，2.33)	<0.001
Age	0.99 (0.99，1.00)	0.016	0.99 (0.98，1.00)	0.011
Race	0.96 (0.87，1.05)	0.363		
Education level	0.80 (0.74，0.87)	<0.001	0.95 (0.86，1.05)	0.005
Marital status	1.11 (1.05，1.17)	<0.001	1.03 (0.97，1.09)	0.397
PIR	0.41 (0.33，0.51)	<0.001	0.66 (0.51，0.86)	<0.001
General health condition	2.85 (2.41，3.38)	<0.001	2.43 (2.00，2.94)	<0.001
Sleep hours	0.85 (0.71，1.01)	0.072		
BMI	1.22 (1.07，1.39)	0.004	1.11 (0.96，1.28)	0.025
Alcohol drinking	0.87 (0.69，1.09)	0.217		
Smoking	1.35 (1.19，1.53)	<0.001	1.17 (1.02，1.35)	0.047
Hypertension	0.72 (0.59，0.89)	0.002	0.86 (0.67，1.10)	0.062
Diabetes	0.70 (0.53，0.91)	0.007	1.06 (0.77，1.46)	0.758
Asthma	0.54 (0.42，0.69)	<0.001	0.71 (0.54，0.94)	0.011
Coronary heart disease	0.70 (0.47，1.06)	0.091		
OSAHS severity	1.25 (1.10，1.41)	<0.001	1.18 (1.03，1.35)	0.024

PIR, ratio of family income to poverty; BMI, body mass index.

**Figure 4:**
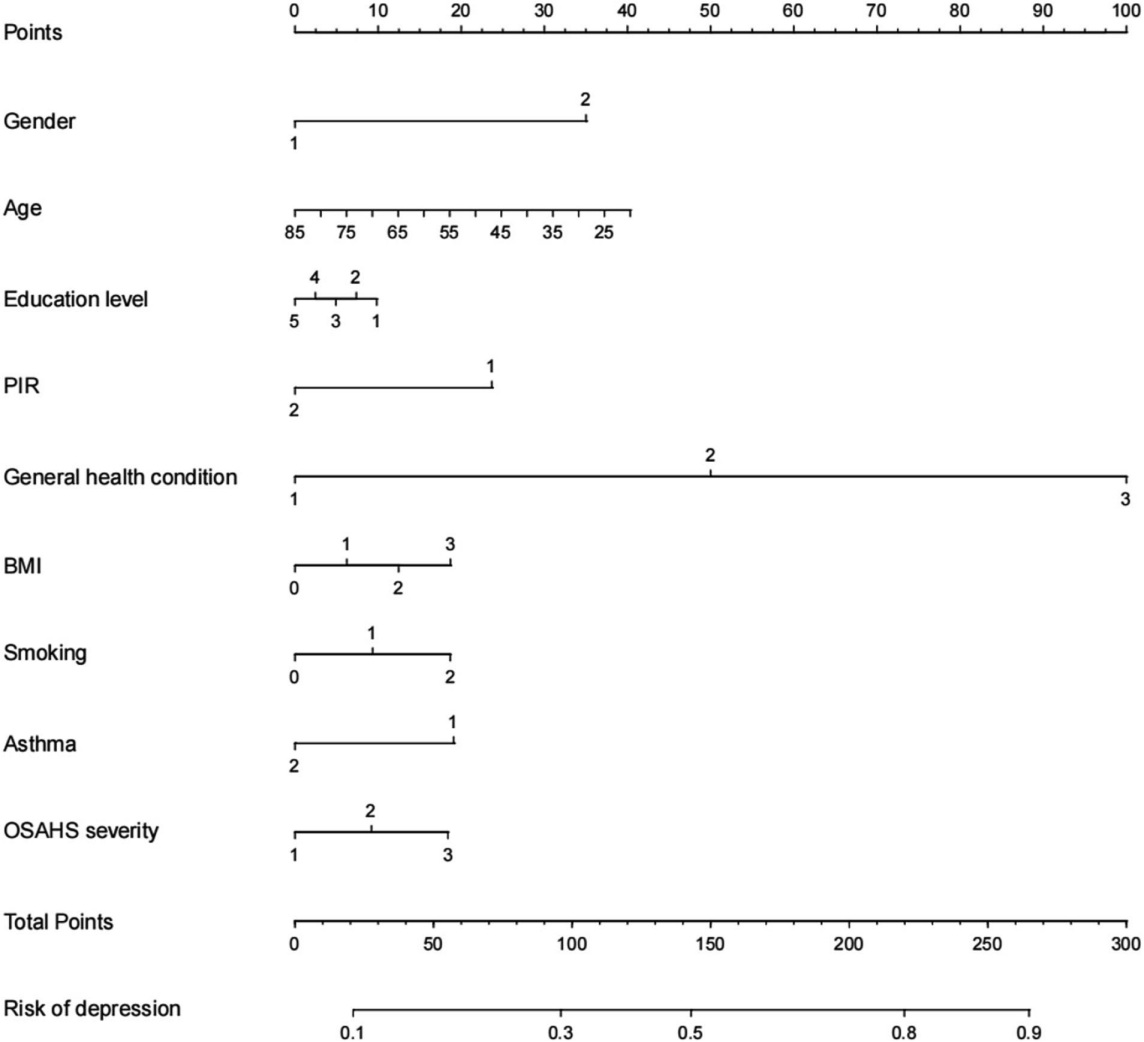
Nomogram prediction model for logistic risk of depression in OSAHS patients. PIR, ratio of family income to poverty; BMI, body mass index.

Based on the factors included in the above regression analysis and the corresponding regression coefficients of each element, a risk prediction model for depression in OSAHS patients was constructed, and a nomogram was drawn. According to the influencing factors in the nomogram and the corresponding scores of each variable, the prediction probability corresponding to the total score was the probability of depression in OSAHS patients when the scores were summed. Points are the individual scores, total points are the full scores, and risk of depression is the incidence of depression corresponding to the total scores, as shown in Figure 4. The nomogram assignment method of relevant factors is shown in Table 6.

**Table 6:** Nomogram of relevant factors in the assignment method.

Risk factors	Assignment
Gender	“Male” = 1, “Female” = 2
Age	Original value entry
Education level	“Less than 9th grade” = 1, “9-11th grade” = 2, “High school graduate” = 3 ，“Some college or AA degree” = 4, “College graduate or above” = 5
PIR	“Low-income” = 1, “Non-low-income” = 2
General health condition	“Good” = 1, “General” = 2，“Bad” = 3
BMI	“Underweight” = 0, “Normal weight” = 1，“Overweight” = 2, “Obese” = 3
Smoking	“Never smoker” = 0, “Former smoker” = 1，“Now smoker” = 2
Asthma	“Yes” = 1, “No” = 2
OSAHS severity	“Mild” = 1, “Moderate” = 2，“Severe” = 3

PIR, ratio of family income to poverty; BMI, body mass index.

#### LASSO model

**Figure 5:**
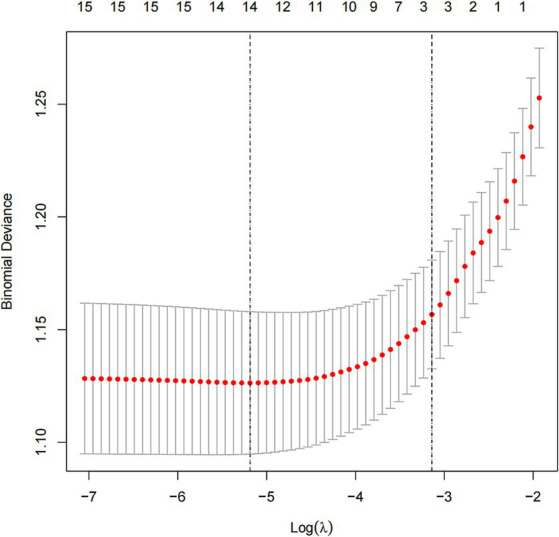
Lasso model ten-fold cross-validation method to screen predictors process diagram.

Depression was used as the dependent variable, and a total of gender, age, race, education level, marital status, PIR, general health condition, sleep hours, BMI, alcohol drinking, smoking, hypertension, diabetes, asthma, coronary heart disease, and OSAHS severity, a total of 16 independent variables. From Figure 5, the optimal model was obtained by selecting the λ value with the minor error (0.005586744) through ten-fold cross-validation. On this basis, we choose the associated with OSAHS depression 14 of the most promising of the independent variables, including gender, age, education level, marital status, PIR, general health condition, sleep hours, BMI, alcohol drinking, smoking, hypertension, asthma, coronary heart disease, and OSAHS severity. We conducted the binary logistic regression analysis based on the selection of variables, and the results were obtained. After analysis, it was found that marital status, PIR, general health condition, sleep hours and smoking are the independent influencing factors of depression in OSAHS patients (*p* < 0.05).

#### Random forest model

**Figure 6:**
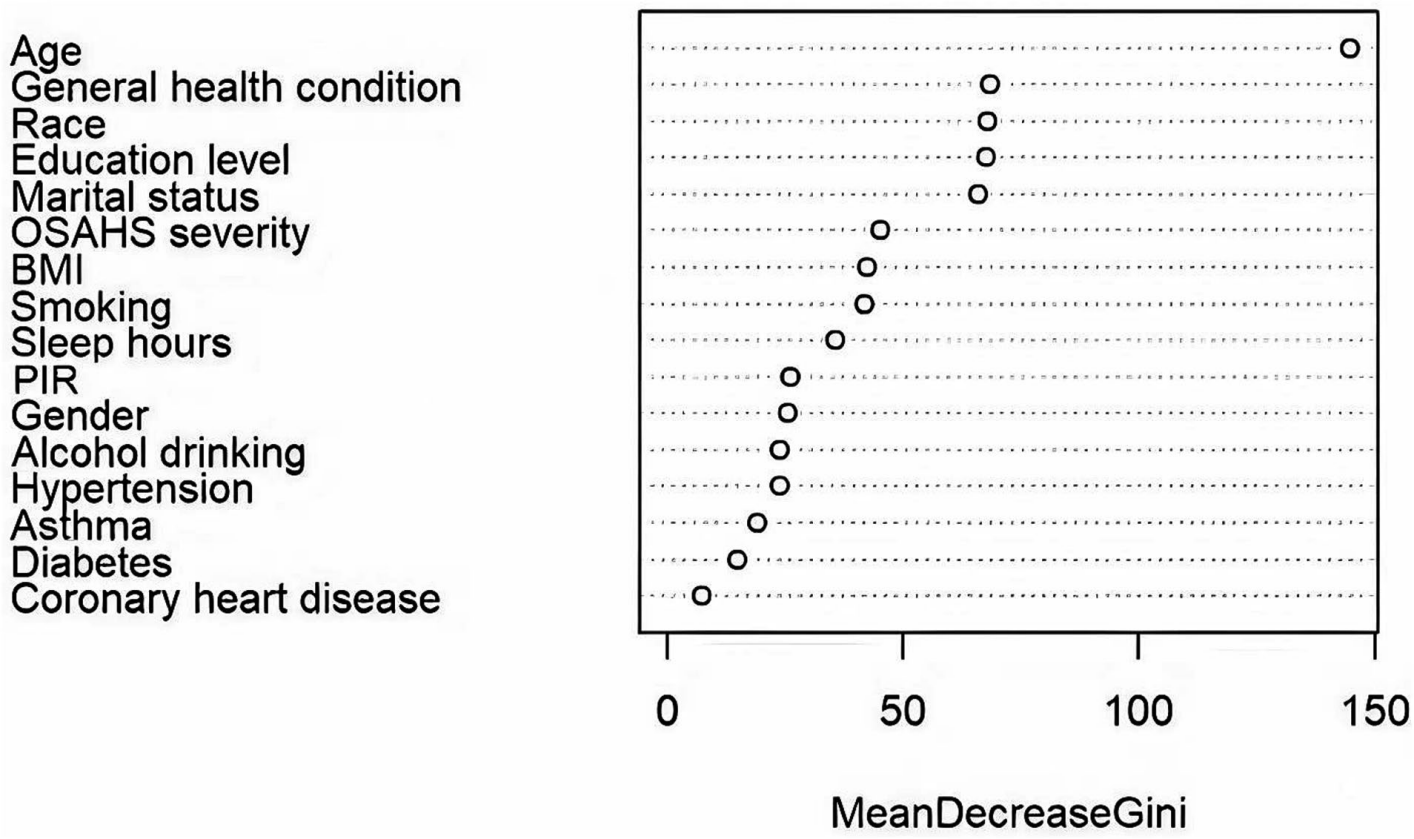
Variable importance plot. PIR, ratio of family income to poverty; BMI, body mass index.

According to the results, age, general health condition, race, education level, marital status, OSAHS severity, BMI, smoking, and sleep hours were the top nine critical factors for predicting depression in OSAHS patients. Figure 6 demonstrates the ranking of importance of these indicators. Subsequently, the above nine variables in binary logistic regression analysis, the final results showed that marital status, general health condition, and smoking were independent influencing factors for depression in OSAHS patients (*p* < 0.05).

### Comparison of model prediction performance

#### Comparison of ROC curve prediction performance

**Figure 7:**
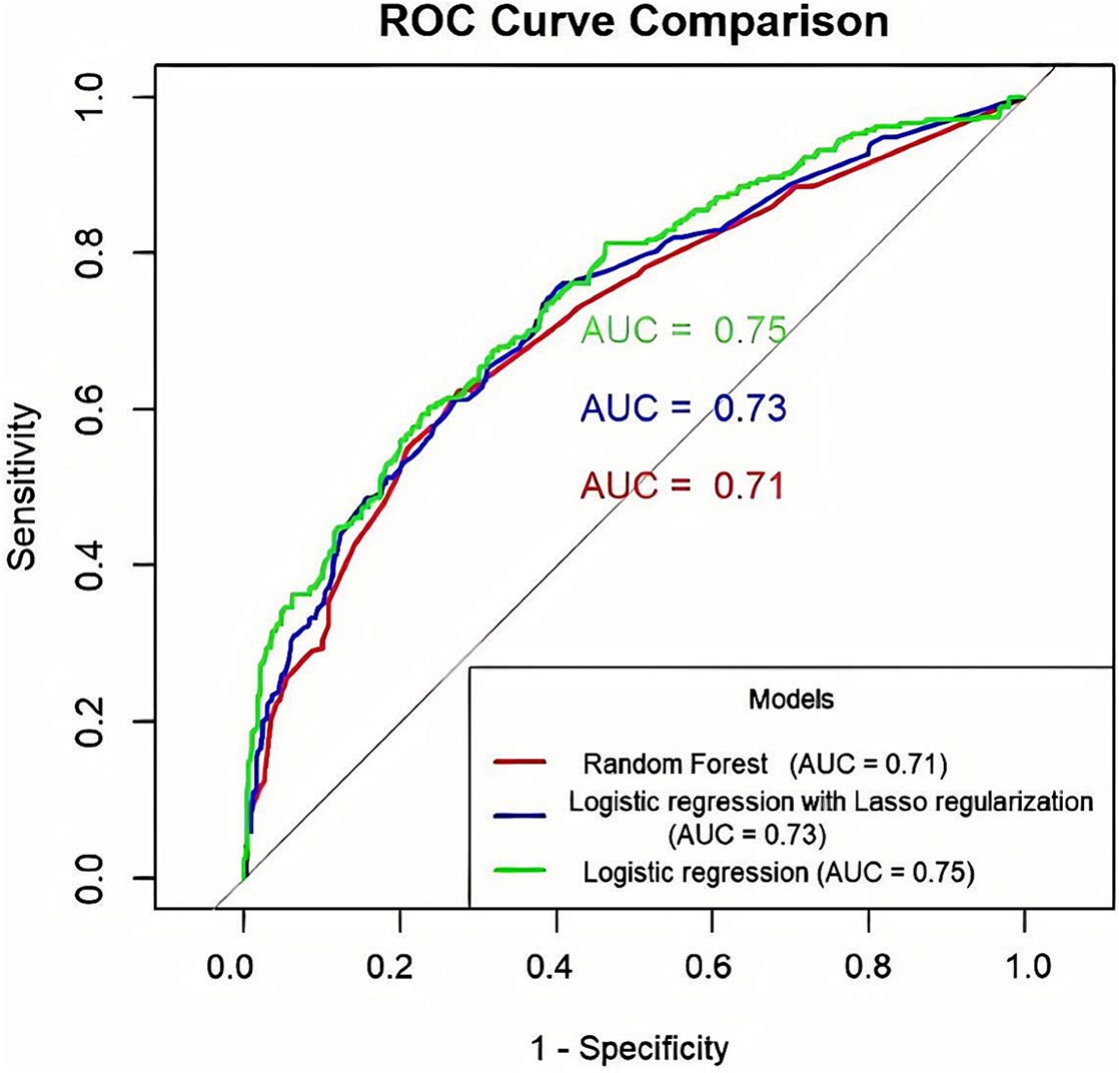
Comparison of ROC curve prediction performance of three prediction models for OSAHS patients with depression (The x-axis indicates the false positive rate, and the y-axis represents sensitivity.).

To compare the performance of the three models in predicting depression in OSAHS patients, we used the test set data for evaluation. Results show that compared to the lasso model, the sensitivity of the logistic regression model is low, but its specificity and AUC area are higher. This means that the logistic regression model performs better in accurately identifying non-depressed patients, while the lasso model is more sensitive in capturing depressed patients. AUC was used as the preferred index to judge the model’s prediction performance. Therefore, the prediction performance of the logistic regression model was better than that of the lasso and random forest models. The comparative results are shown in Table 7. The ROC curve is shown in Figure 7.

**Table 7:** Comparison of prediction performance of three kinds of models.

Model	AUC	95%CI	Sensitivity	Specificity	Youden index
Random forest	0.710	(0.669, 0.752)	0.624	0.727	0.350
Lasso	0.727	(0.687, 0.767)	0.756	0.599	0.355
Logistic regression	0.746	(0.707, 0.785)	0.603	0.764	0.367

#### Comparison of DCA prediction performance

**Figure 8:**
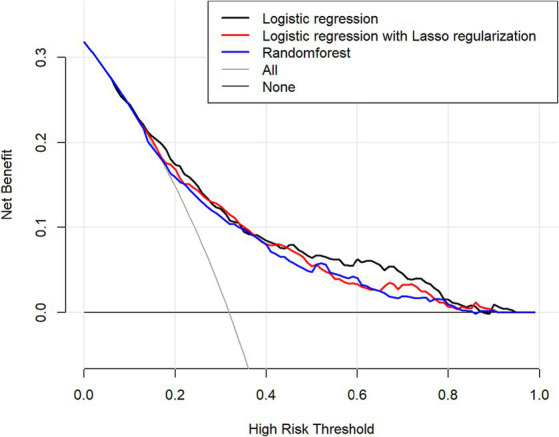
Comparison of the predictive performance of three predictive models decision-making curve analysis (DCA) predictions (The x-axis indicates the high risk threshold, and the y-axis represents net benefit.).

Clinical decision curve analysis of the prediction model found that when the probability threshold was in the range of 0.19 to 0.82, the prediction model had an excellent net benefit in predicting depression in OSAHS patients. The decision curve analysis results show that the net benefits of the three models were similar for thresholds probability ranging from 0.25 to 0.45. When the threshold probability range was 0.19–0.25 and 0.45–0.82, respectively, the net benefit of the logistic regression model was the most significant. Therefore, the logistic regression model showed better clinical utility than the random forest and lasso models as shown in Figure 8.

Considering the above indicators, the logistic regression model has better predictive performance than the lasso and random forest models in predicting depression in OSAHS patients. And analysis of the influence of related factors, including gender, general health condition, BMI, smoking, OSAHS severity, age, education level, PIR, and asthma.

#### Clinical utility

**Figure 9:**
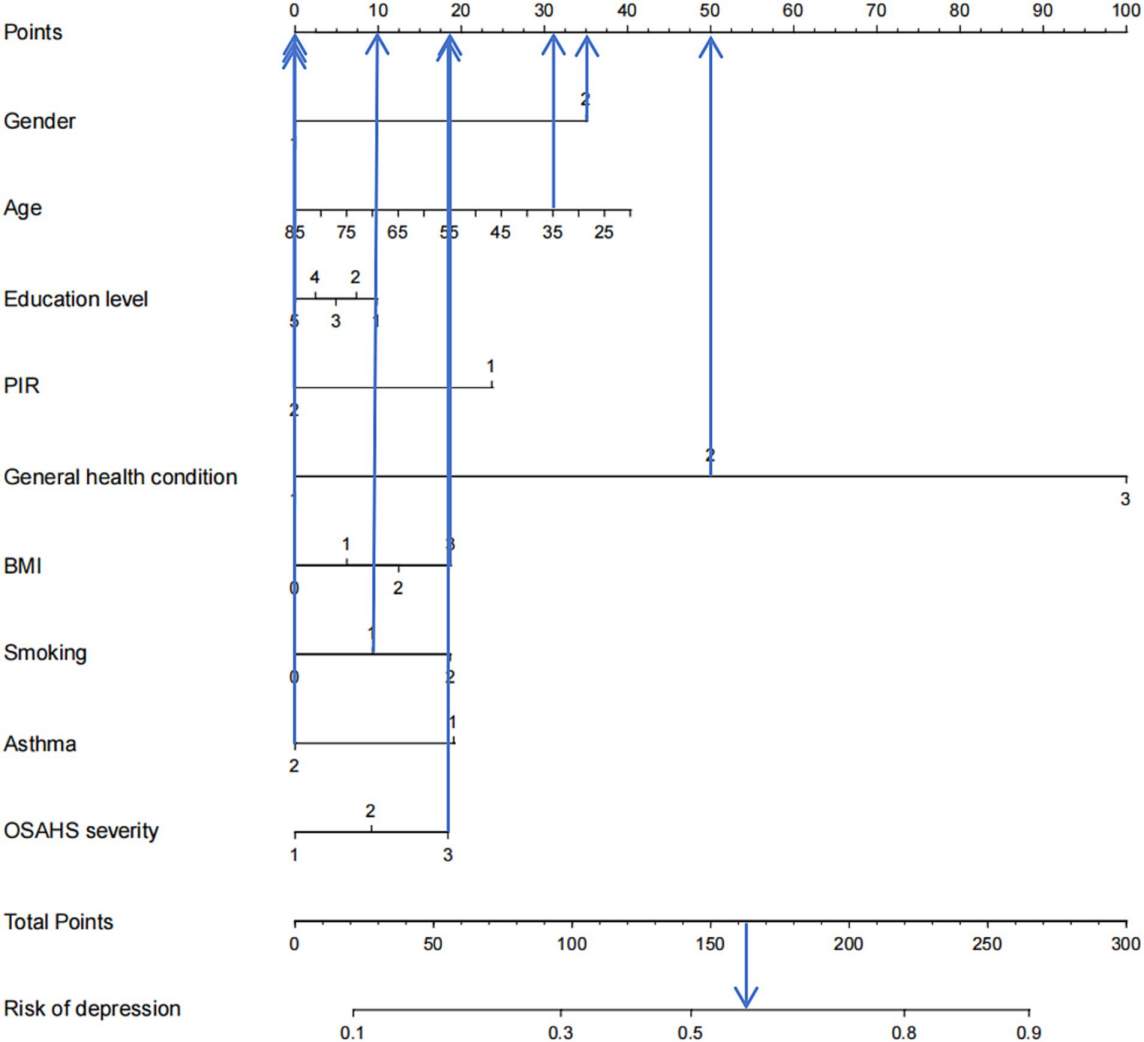
Example of nomogram. PIR, ratio of family income to poverty; BMI, body mass index.

Figure 9 shows an example of a patient’s nomogram. The patients who are 35 years of age, female, have a bachelor’s degree, low income, have general health, obesity, and smoking in the past, now give up smoking, do not admit to a history of asthma, suffer from severe OSAHS. According to the diagram model, the patients with a total score of 163.5 points have a probability of about 57% of depression.

## Discussion

The main strength of this study is the use of the NHANES extensive sample database and the use of ML models to predict and identify potential influencing factors of depression in OSAHS patients. In this study, we constructed and trained a model based on the depression of OSAHS adults in the US. We considered variables such as demographic characteristics, lifestyle, and health factors and weighted them during the construction of the model. Through our selection model and can be output in probability of OSAHS adult depression in the United States. These results improve the intelligence of the mental health care system and have a positive impact. Sleep-related healthcare providers can use these ML algorithms to identify OSAHS patients who are potentially at risk for depression, which in turn can detect early if they are suffering from depression and help them with early intervention. The research also further increases the chances of OSAHS patient’s access to mental health services. This study will explore the incidence of depression in American adults with OSAHS, the influencing factors, and the predictive power of risk prediction models. The practical application of these findings to real life will also be discussed.

### Depression among OSAHS adults in the US

The present study was based on three cycles of NHANES data (2005–2006, 2007–2008, and 2015–2016) and included variables that included demographic characteristics, lifestyle, and health information. A total of 2,453 OSAHS patients were screened, including 1,671 non-depressed patients and 782 depressed patients. The incidence of depression was 31.9%, slightly lower than the findings of Houda Gharsalli and Siddharth Bajpai et al. (4, 13). The difference in the incidence of depression may be due to the different inclusion criteria used in the OSAHS population. The study by Houda Gharsalli and Siddharth Bajpai et al. employed specialized diagnostic equipment, such as polysomnography (PSG), with an apnea-hypopnea index (AHI) ≥5 as the diagnostic criteria for OSAHS. In contrast, this study was judged and included only based on patients’ self-reported results on “How often do you snore/stop breathing?.” At the same time, temporal and geographic differences may also contribute to lower rates of depression in the OSAHS population than the results of other studies.

### Factors influencing the development of depression in OSAHS adults in the US

This study used three ML algorithms, logistic regression, lasso, and random forest, to construct a predictive model of depression in the US OSAHS population. Results show that the logistic regression model is better than the random forest and logistic regression models in terms of specificity, Youden index, and AUC. However, its sensitivity is lower. Therefore, according to the results of this study, among the three prediction models, the logistic regression model performs better than the lasso model and the random forest model. In predicting the occurrence of depression in the OSAHS population, the model is affected by factors such as gender, age, education level, PIR, general health condition, BMI, smoking, asthma, and OSAHS severity. In addition, the model results are visually presented in Figure 4 in this study.

In terms of sociodemographic characteristics， according to the findings of Alimohamad Asghari, Magali Saint Martin, and Yaozhang Dai, as well as Rachel H. Salk, women are more prone to depression relative to men (35–37). This difference exists not only in China but also in most countries and cultures worldwide. In general, women are twice as likely to suffer from depression (OR = 1.95) than men, which may be related to frequent hormonal disturbances due to genetic and physiological factors. Some studies have shown that women are more likely to experience depression during menopause (38, 39). According to the results of this study, obese individuals are more likely to suffer from depression compared to normal weight or underweight individuals, which is consistent with the findings of Ashley Wendell Kranjac and Tuula H. Heiskanen (40, 41). According to Ashley Wendell Kranjac et al., after taking into account the combined effects of gender and BMI, obese women were 43% more likely to experience depression than normal-weight women. Tuula H. Heiskanen’s study, a 6-year prospective study of outpatients, found that subjects with significant weight gain were more likely to develop major depression (41). This may be because obese people often suffer from associated chronic inflammation. Immune cells in adipose tissue produce signaling proteins related to inflammation, and some of these proteins, such as cytokines, are strongly related to mental health problems and have even been used as biomarkers for depression (42).

Compared to older people, teens are at higher risk of depression. This is consistent with the findings of Stephanie Wagner (43). In the study of Stefanie Wagner, hospitalized patients with depression were divided into four different age groups. The results showed that the aged 18 to 29 years old young patients are more likely to show extreme behavior, such as suicide and drug abuse. In contrast, middle-aged and older patients aged between 50 and 65 years were more likely to show mild depression, such as decreased sexual interest. This may be related to adolescent adolescence body hormone disorder and lack of mental toughness, leading to mental instability (44).

The higher the level of education, the lower the risk of depression. Early studies have pointed out that depression is associated with low levels of education (45), and education has a significant effect on the development of depression. That is, illiterates are more likely to have more severe depression (46). This may be due to the low level of education, which leads to narrower social contacts and fewer avenues for problem solving when experiencing negative life events and is more likely to cause heavier negative emotions, which can lead to depression.

The findings suggest that low-income people are more likely to suffer from depression than non-low-income people, which was confirmed in the study of Glaesmer (47). It is estimated that while around half of people with depression in high-income countries are not diagnosed or treated, in low-income and middle-income countries, the proportion may be as high as 80%–90%. According to a December 2020 commentary in the journal *Science*, there is a causal interaction between poverty and mental illness. This means that people with low incomes are more vulnerable to the threat of mental illness, and at the same time, mental illness is also one of the vital causes of people with low incomes (13). This phenomenon may be because low-income people usually face financial difficulties in their daily lives and lack sufficient funds to meet basic needs such as food, housing, and healthcare. This financial pressure may make them feel helpless, anxious, depressed, and more prone to depression.

The poorer the general health condition, the greater the probability of the risk of depression. This finding is consistent with results from the World Health Survey published by Saba Moussavi in The *Lancet*. This study showed that depression was associated with the lowest health scores, both in isolation and in co-occurrence with other chronic diseases (48). A research study by Nicolas Zdanowicz also indicated that physical health and its improvement are related to the level of depression (49), and Érica Dorigatti de Ávila clarified that patients without depression have a higher level of mental health (50). The possible cause is physical factors. Bad health condition is accompanied by chronic illness, pain, or discomfort that may affect an individual’s emotional and psychological state, thereby increasing the risk of depression. In addition, due to limited physical function and lower quality of life, people with depression may harm their self-worth and self-esteem. This psychological stress may further aggravate depression. Chronic physical illnesses and health problems may cause individuals to develop negative feelings and increase the risk of depression. And overall, poor health increases the personal burden of life. People may need more time and money to treat their diseases, which may lead to economic stress and anxiety, thus increasing the risk of depression.

According to the study, smoking is considered to be one of the factors that predict the high risk of depression in OSAHS patients. Specific studies have shown higher rates of depression among current and former smokers compared with never smokers. According to a survey of the U.S. population, LUIS G. ESCOBEDO found that former smokers are more likely to develop depression, especially those who have a history of major depression (51). The cross-sectional study conducted by Tana M. Luger also noted that current smokers were more likely to develop depression than never-smokers. In contrast, current smokers were more likely to develop depression than former smokers (52). This phenomenon may be because nicotine intake from smoking can bring short-term pleasure and relaxation. Still, long-term smoking may lead to neurotransmitter disorders, thereby affecting emotional stability and increasing the risk of depression.

People with asthma are more likely to suffer from depression than people without asthma. This idea is supported by a biological linkage study by Mingdi Jiang et al. It implies that the inflammatory response may be a critical factor in regulating the common pathways of depression and asthma (53). The results of Mahima Akula’s study also confirmed the correlation between the two (54). Furthermore, a bidirectional association between asthma and depression was observed in Hyo Geun Choi’s study (55). In a clinical practice study of adolescents with asthma, 11.5% had depression (42). This may be because people with asthma may feel negative emotions such as low self-esteem, anxiety, and fear. Due to asthma having wave properties, some patients may need to avoid social situations and activities, which may lead to individual patients being isolated and isolated, which will affect their psychological health. In addition, patients with asthma often face physical discomfort such as dyspnea and chest tightness, which may affect the individual’s emotional and psychological state, which in turn exacerbates negative emotions and increases the risk of depression.

As the severity of OSAHS increases, so does the risk of depression. Cass Edwards et al. Research confirmed the results and found that with the rise in the severity of OSAHS, PHQ score and the incidence of depression also gradually increased (56). This result may be due to the OSAHS patients during apnea oxygen supply is insufficient and hypoxemia. With the deterioration of OSAHS, hypoxemia has a more serious negative impact on brain function and emotion regulation, which leads to the occurrence of depression. In addition, patients with OSAHS may experience symptoms such as fatigue, lethargy, and difficulty concentrating during the day due to decreased sleep quality. These limitations in daily functioning may negatively affect an individual’s psychological state and increase the risk of depression.

Severe OSAHS can also cause sleep disturbances, which in turn can lead to a decline in social activities and work ability. This situation is further exacerbated by negative emotions such as anxiety, low self-esteem, and depression. Therefore, it can be concluded that there is a strong correlation between OSAHS and depression and that its severity is positively related to the risk of depression.

### Evaluation and application of risk prediction models for depression

Research results show that the logistic regression model is better than the random forest and lasso models regarding specificity, Youden index, and AUC area. To validate the model in the clinical application value of this study, the DCA was used, and the net income of the model was used in the comparison. The results showed that the logistic regression model had the most significant net benefit within the vast majority of threshold probability ranges (0.19 to 0.25 and 0.45 to 0.82) and had a good effect on clinical application. Therefore, the comprehensive prediction ability of the comparison results shows that the logistic regression model is superior to the lasso and random forest models. It should be noted that the lasso model may ignore some relevant features due to the high correlation between features. In addition, selecting the appropriate regularization parameter needs experience, cross validation, and other methods. This increases the complexity of model tuning (57). The random forest model is composed of multiple decision trees. Although feature importance can be used to understand the contribution of each feature to the model, the overall model is less explanatory than the logistic regression model. In addition, due to the random forest model to build a decision tree and perform multiple feature selection and integration of the operation, its training time is relatively long (58). In contrast, the logistic regression model, as a generalized linear model, uses the least squares method to fit the model and thus has high accuracy (59). Logistic regression models can make predictions and explore the direction and degree of influence between independent and dependent variables, so they have better explanatory power (49). Logistic regression models can be quantified and visualized by nomograms, which have outstanding advantages in auxiliary diagnosis in the medical field (60).

Before applying a risk prediction model for depression in the OSAHS population, it is necessary to select the most appropriate model and adjust the parameters to maximize the prediction effect of the model to improve the accuracy and sensitivity of identifying OSAHS patients at high risk for depression. Subsequently, predictive models need to be translated into forms applicable to the community and clinic, such as nomograms or mobile applications that allow physicians and sleep technologists to calculate the probability of depression easily and quickly. This will help to protect OSAHS patients in advance and effectively prevent the occurrence of depression.

In this study, ML models, especially logistic regression models, demonstrated excellent depression prediction and recognition capabilities in a large dataset. In contrast to traditional statistical methods, ML methods no longer require the researcher to specify the relevant variables subjectively but can automatically identify the variables associated with the outcome variables in the data set. This is precisely one of the advantages of ML in building clinical prediction models. Future research could apply ML methods to model and compare in longitudinal studies to obtain basic information such as the prevalence of depression. For example, as far as studies in predicting depression are concerned, studies like those done by Dai Su et al. in a longitudinal study of the older adult population in China are a good example (20). In addition, it is possible to cross-combine multiple models in ML to form a hybrid model and verify whether the hybrid model outperforms the traditional single ML model in terms of predictive performance (61). To better psychological doctors and health care at all levels, provide appropriate information and services.

In a clinical sense, physicians can assess whether a patient is at risk for depression based on gender, general health condition, BMI, smoking, OSAHS severity, age, education level, PIR, and asthma. Once a patient is identified as being at risk for depression, interventions can be implemented, including medication, psychotherapy, and behavioral changes. Early intervention and treatment can help patients reduce the symptoms of depression, improve their sleep quality and quality of life, and improve the effect of OSAHS treatment.

When using the logistic regression model to predict depression in OSAHS patients, this study suggests that specificity, sensitivity, and Youden index should be considered comprehensively, and the choice of specificity and sensitivity should be weighed according to the specific situation. In addition, in clinical practice, demonstrating the effectiveness of these indicators is also necessary. To ensure the applicability and reliability of the model, it is recommended to continue to collect larger scale, diverse data and use these data to validate and replicate the findings. By expanding the dataset’s scope, the model’s predictive performance in different populations and contexts can be more fully evaluated. Such efforts can help improve the accuracy and generalization ability of the model and provide a more reliable basis for future clinical practice. When the model is applied in clinical practice, it needs to be comprehensively evaluated by combining clinical experience and individual patient differences. This process involves interpretation and interpretation of the model predictions and a comprehensive consideration of the patient’s situation. At the same time, it is necessary to continuously update and optimize the model to improve its predictive performance and clinical utility. Finally, for the research on the influencing factors, the specific mechanism of each factor’s influence on the occurrence of depression can be further explored in depth. This includes detailed studies of biological, psychosocial, and other factors to reveal their associations with depression. At the same time, how to prevent and treat depression by intervening in these factors can also be studied.

The results of this study can be incorporated into the development and implementation of relevant public health policies. Government departments can develop prevention and intervention strategies for depression in OSAHS patients according to the logistic regression model and influencing factors identified in this study. By formulating strategies based on these models and influencing factors, patients’ mental health can be effectively improved. Department of Public Health and medical institutions can reasonably allocate resources to strengthen the prevention and treatment of depression in patients with OSAHS. This could include making more counselors or psychotherapists available and improving screening and diagnostic facilities for depression, among others. In related health education activities, the findings of this study should be disseminated to improve the public’s awareness and attention to depression in OSAHS patients. This helps reduce the social discrimination against depression to promote social support and understanding. It can also guide medical practice. By applying a logistic regression model, the medical personnel can more accurately identify the existence of the risk of depression in patients with OSAHS, which can carry on the intervention and treatment in a timely manner. This method helps to improve the early diagnostic rate of depression and to provide more effective personalized treatment options for patients to enhance their mental health. On prevention strategies, the results of this study are to develop in OSAHS patients with depression prevention strategy provides an essential basis. Based on the logistic regression model, the influence factors of medical personnel can be targeted to carry out the intervention measures, including strengthening the OSAHS patients’ psychological health education and psychological support services and establishing health management programs.

It should be noted that our results may have been affected by various potential biases in the NHANES database. One of the main biases is the low participation rate of specific populations, which may introduce sampling bias. Therefore, the data representation may be poor and cannot fully reflect the OSAHS group. This may affect the generalizability of the findings. Therefore, it is suggested that a multicenter study be carried out to expand the sample representativeness and increase the external data validity and generalization ability. Second, it should be noted that some of the data in the NHANES database rely on participant self-reports, such as diagnostic information for patients with OSAHS. This dependence may be limited by the deviation of subjective evaluation and memory bias, the influence of such factors to assess the severity of OSAHS symptoms, or inaccuracy. This may affect the reliability and accuracy of the findings. Therefore, to improve the objectivity and accuracy of diagnosis, this study suggested introducing objective measurement tools, such as more sleep figures (PSG). Using these objective measurement tools can reduce the dependence on self-reported respondents and help assess the symptoms and severity of OSAHS more accurately. Suggestions in the study consider integrating other data sources, such as clinical records and medical insurance databases, to get more comprehensive and multi-angle OSAHS data. By combining these diverse data sources, the reliability and generalizability of the findings can be increased.

In addition, future research should further optimize the ML model’s performance to improve the prediction accuracy of depression in patients with OSAHS. The introduction of other machine learning algorithms or deep learning methods can also be considered to explore better predictive models. These methods can model and analyze the data from different perspectives and improve the stability and accuracy of the model. By exploring effective intervention strategies and conducting intervention trials, the effects of other methods can be evaluated, and their long-term effects can be followed up to reduce the incidence of depression and improve the quality of life of patients with OSAHS.

## Conclusion

This research uses the NHANES database to establish three ML models, the logistic regression model, the lasso model, and the random forest model, to predict depression in the OSAHS group and identify the related factors. Among them, the logistic regression model was superior to the lasso and random forest models’ overall prediction performance. By drawing the nomogram and applying it to the sleep testing center or sleep clinic, sleep technicians and medical staff can quickly and easily identify whether OSAHS patients have depression to carry out the necessary referral and psychological treatment.

## Limitations

The data used in this study were obtained from the NHANES database, which includes a variety of relevant variables, such as smoking history and sleep disorders. Most of these variables are based on patient self-reporting and may have subjective bias, affecting the data’s objective accuracy.

Due to the lack of relevant variables in the NHANES database, such as the AHI index, minimum oxygen saturation, etc., including these predictors may allow for better model prediction performance.

In this study, due to the limited sample size of the screening, we did not perform external validation to verify the effect of this predictive model. However, we hope that future studies will externally validate this model by conducting a multicenter study with an increased sample size and applying it to a community population for screening further to test the generalization ability and robustness of the model.

## Data availability statement

The raw data supporting the conclusions of this article will be made available by the authors, without undue reservation.

## Ethics statement

The NCHS Research Ethics Review Board (ERB) reviewed and approved the studies involving human participants. Written informed consent for participation was not required for this study by the national legislation and the institutional requirements.

## Author contributions

EL: Writing – original draft, Conceptualization, Data curation, Formal analysis, Resources, Visualization. FA: Writing – review & editing, Investigation, Methodology, Software. CL: Writing – review & editing, Funding acquisition, Project administration, Supervision, Validation.

## Footnotes

^1^ https://www.cdc.gov/nchs/nhanes/index.htm
