# Supplementary material for: A machine learning model to predict the risk of depression in US adults with obstructive sleep apnea hypopnea syndrome: a cross-sectional study
Source: Front Public Health. 2024 Jan 8;11:1348803. doi: 10.3389/fpubh.2023.1348803 (PMC10800603; doi:10.3389/fpubh.2023.1348803)
Supplement: Supplementary file 1 [file Data_Sheet_1.PDF]

Nomogram model of depression in OSAHS patients

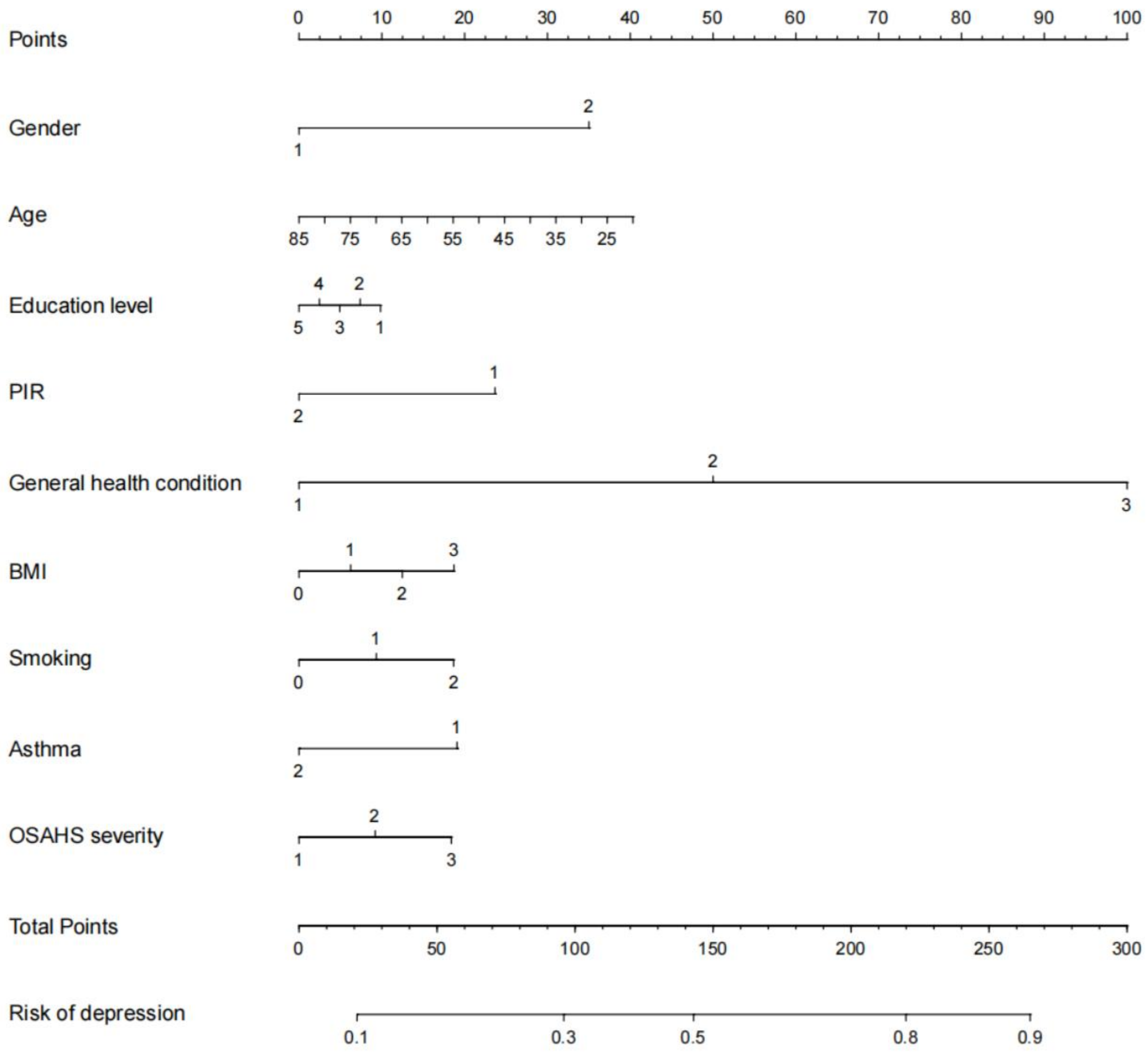

PIR：ratio of family income to poverty ， BMI：body mass index.

<< The methods for assigning the relevant factors in the nomogram are shown in the table on the next page. >>

Methods for assigning relevant factors in nomogram

Risk factors	Assignment
**Gender**	"Male" = 1, "Female" = 2
**Age**	Original value entry
**Education level**	"Less than 9th grade" = 1, "9-11th grade" = 2, "High school graduate" = 3，"Some college or AA degree " = 4 ， "College graduate or above" = 5
**PIR**	"Low-income" = 1, "Non-low-income" = 2
**General health condition**	"Good" = 1, "General" = 2，"Bad" = 3
**BMI**	"Underweight" = 0, "Normal weight" = 1， "Overweight" = 2, "Obese " = 3
**Smoking**	"Never smoker" = 0, "Former smoker" = 1，"Now smoker" = 2
**Asthma**	"Yes" = 1, "No" = 2
**OSAHS severity**	"Mild" = 1, "Moderate" = 2，"Severe" = 3

PIR：ratio of family income to poverty ， BMI：body mass index.
